# Personality factors and pandemic-related behaviors

**DOI:** 10.3389/fpsyg.2024.1389672

**Published:** 2024-07-31

**Authors:** Jessica Williamson

**Affiliations:** Department of Psychology, California State University, Bakersfield, Bakersfield, CA, United States

**Keywords:** COVID-19, compassion for others, narcissism, psychopathy, personality

## Abstract

**Purpose:**

The purpose of the current study was to determine whether there are personality differences (the HEXACO model, narcissism, sadism, compassion for others) in mask-wearing, social distancing, and hoarding.

**Findings:**

Those who always wore masks were significantly higher in compassion for others and significantly lower in sadism compared to those who did not always wear masks. Those who always socially distanced (compared to those who did not) were significantly higher in openness, compassion for others, and conscientiousness. Those who hoarded were significantly lower in agreeableness than those who did not hoard.

**Conclusion:**

Perhaps physicians may use information to boost states of altruistic-type traits (agreeableness, compassion for others) while educating patients during visits in order to increase the likelihood of receiving vaccinations or booster shots.

## Introduction

The COVID-19 pandemic thus far has claimed the lives of over 6,600,000 people globally since it began (Mathieu et al., 2022). Thus far it is ranked 7th in a list of 19 of the deadliest known pandemics, surpassed only by the Bubonic plague, smallpox, the Spanish Flu, the Plague of Justinian, HIV/AIDS, and the Third Plague (LePan, 2023).

Since the start of the pandemic, researchers have assessed traits from the “Big 5” as potential predictors of certain pandemic related behaviors and outcomes. The Big 5 is a taxonomy that refers to a set of traits deemed as cross-culturally important (Digman, 1990) and consists of openness/intellect/culture, conscientiousness, extraversion, agreeableness, and neuroticism/emotional stability. Openness/intellect/culture can be conceptualized as the propensity of an individual to create original and complex ideas, to enjoy and seek out novel experiences, and to value diversity (John et al., 2008). Conscientiousness refers to impulse control, one’s ability to stay on task, one’s organization skills, ability to set goals and plan ahead, observation of and adherence to societal rules and norms, and ability to postpone gratification when necessary (John et al., 2008). Extraversion refers to one’s level of positive emotions, sociability, outgoingness, energy, and assertiveness (John et al., 2008). Agreeableness refers to one’s propensity toward tendermindedness, trust, and lack of antagonism (John et al., 2008). Finally, neuroticism refers to emotional stability, levels of negative emotionality, propensity toward anxiety, sadness, tension, and nervousness. More recently, researchers (Ashton et al., 2004; Ashton and Lee, 2009) have come up with a possible sixth universal trait – honesty-humility. Honesty-humility is the propensity toward fairness, modesty, sincerity, and greed avoidance (Ashton and Lee, 2009; Lee and Ashton, 2013). Emotionality in the HEXACO model is also somewhat different from neuroticism in many other Big 5 measures – it has much of the same content that neuroticism (in the Big 5) has, but it lacks the anger-related components of neuroticism and includes the sentimentality component of the Big 5’s agreeableness factor.

Han (2021) found that, with the exception of extraversion, higher levels of all Big 5 traits positively predicted individuals’ propensity to engage in social distancing and to comply with general protective measures during the pandemic. Similarly, Krupić et al. (2021) found that higher levels of agreeableness and conscientiousness predicted greater adherence to preventative measures such as social distancing and mask-wearing. Airaksinen et al. (2021) also found that people with higher levels of openness/intellect/culture, conscientiousness, and neuroticism/emotional stability were more consistent in mask-wearing and social distancing among older adults. Xie et al. (2020) found that factors such as agreeableness can influence mask-wearing and social distancing. Carvalho et al. (2020) have also found that higher levels of extraversion predict lower levels of social distancing, whereas higher levels of conscientiousness predict a greater likelihood of social distancing. Götz et al. (2021) examined over 100,000 participants in over 55 countries and found that openness/intellect/culture, conscientiousness, agreeableness, and neuroticism/emotional stability predicted a greater likelihood of sheltering-in-place whereas higher levels of extraversion were related to a decreased likelihood of sheltering-in-place. When using the HEXACO model, Costantini et al. (2022) found that honesty-humility was the strongest predictor of engaging in safer behaviors such as social distancing. Researchers conducting a meta-analysis (Zettler et al., 2022) found that emotionality/neuroticism/emotional stability from the HEXACO was related to greater anxiety about health concerns and that those higher in narcissistic-type traits were not as willing to accept quarantine and social-distancing restrictions.

Certain traits, such as narcissism and sadism, are related to callous behaviors that indicate a general lack of care and consideration of the wellbeing of other people (Paulhus and Williams, 2002; O'Connell and Marcus, 2019). Both sadism and narcissism are part of the Dark Tetrad (narcissism, Machiavellianism, psychopathy, and sadism; Buckels et al., 2013). Narcissism is generally defined as having a grandiose view of the self and one’s importance as well as an exaggerated sense of entitlement (Paulhus and Williams, 2002) while sadism is defined as taking pleasure in the suffering of others and the desire to dominate others (O'Connell and Marcus, 2019). Traits such as narcissism and sadism my explain why some people refuse to engage in protective measures or steps to ensure the comfort and safety of others. People high in these traits may feel entitled to scarce goods (e.g., hoarding essential items) and they perhaps do not care about the health and wellbeing of others (as evidenced by not wearing masks or socially distancing). They may also take pleasure in causing others distress by purposefully not wearing masks or socially distancing.

Research conducted earlier in the pandemic (Triberti et al., 2021) on dark traits such as narcissism has found that higher levels of these traits were related to a lesser likelihood to sanitize or disinfect commonly touched objects and wash hands during the beginning of the pandemic. Furthermore, higher levels of dark traits are also related to less compliance in mask-wearing (Chávez-Ventura et al., 2022). Sadism has also been shown to be a predictor of refusal to be vaccinated for COVID-19 (Li and Cao, 2022).

On the opposite end of dark traits would be traits related to empathy and compassion for others (Marsh, 2011). As a trait, compassion for others is characterized by a desire or motivation to offer help when witnessing the suffering of others (Goetz et al., 2010). Most (if not all) of the dark types of traits are linked with lower levels of compassion for others (e.g., Marsh, 2011). Behaviorally, exercising compassion for others involves helping others, perhaps even at the risk of exploitation to the self (Goetz et al., 2010). Researchers (e.g., Fazio et al., 2021; Karnaze et al., 2022) have found that higher levels of compassion for others are related to a greater likelihood of staying home during the beginning of the pandemic.

Hoarding during the pandemic has been a less-researched topic. Yoshino et al. (2021) found that levels of agreeableness, neuroticism/emotional stability, and openness/intellect/culture were related to a greater likelihood to hoard in a Japanese sample of participants. There is a paucity of research on the relationship between traits such as compassion for others, honesty-humility, narcissism, sadism, and hoarding behavior relating to the pandemic.

Although there are several studies examining the relationship between pandemic-related behaviors and the Big 5, there are fewer studies examining such behaviors related to the HEXACO model. There are also few studies examining such behaviors related to “dark” type traits such as sadism and narcissism. Sadism and narcissism are distinct from the Big 5 and HEXACO models yet may also offer explanations as to why some individuals may be less likely to engage in safety-related behaviors designed to protect others’ health/.

### Goals and hypotheses of the current study

Based on past research (e.g., Carvalho et al., 2020; Xie et al., 2020; Airaksinen et al., 2021; Götz et al., 2021; Han, 2021; Costantini et al., 2022), it was hypothesized that levels of openness/intellect/culture, conscientiousness, neuroticism/emotional stability, honesty-humility, and agreeableness would be higher and levels of extraversion would be lower in those report always wearing masks and socially distance vs. those who report that they do not always wear masks or socially distance. Those who always wear masks and always socially distance should also be higher in compassion for others compared to those who do not always wear masks or socially distance (Fazio et al., 2021; Karnaze et al., 2022). Those who always wear masks and socially distance should be lower in dark triad type traits such as narcissism and sadism compared to those who do not always wear masks or always socially distance (Triberti et al., 2021; Chávez-Ventura et al., 2022). A major goal of this study is to replicate findings on the Big 5/HEXACO while also expanding upon past research on compassion for others and dark triad type traits as such factors aren’t widely studied in recent pandemic research.

Personality differences in those who did and did not hoard during the pandemic have not been widely examined in past research thus far. Although Yoshino et al. (2021) found that agreeableness, neuroticism/emotional stability, and openness/intellect/culture predicted hoarding behavior, they used only a Japanese sample and did not examine other traits such as honesty-humility, narcissism, sadism, and compassion for others. It was hypothesized in the current study that those who hoard would be higher in neuroticism/emotional stability, openness/intellect/culture, conscientiousness, narcissism, sadism and lower in honesty-humility, agreeableness, and compassion for others compared to those who did not hoard. It was also hypothesized that there would be no significant differences in extraversion related to hoarding status during the pandemic.

Rieger (2020) showed that priming people with messages about how vaccines protect others resulted in individuals showing a greater likelihood of future vaccination (when available) compared to providing messages about vaccines protecting only the self. This type of message might have caused a temporary boost in state levels of traits such as honesty-humility, agreeableness, and sentimentality aspects of emotionality. If findings from this study further show that certain traits play a role in protective behaviors such as socially distancing and masking when sick/contagious, then findings may also be beneficial to healthcare professionals when encouraging patients to receive vaccinations during in-person visits by temporarily boosting state levels of certain traits to get them to agree to vaccinations and boosters during their visit. For example, physicians might educate participants on the risks to strangers and loved ones if the patient is not vaccinated, thereby perhaps temporarily increasing altruistic-type traits (honesty-humility, agreeableness, compassion for others) to increase vaccination and booster compliance. Findings may also inspire future researchers on how to more accurately target messages to encourage people who are sick or contagious to engage in proper social distancing and mask-wearing.

## Materials and methods

### Sample and procedures

Participants were 455 university students from a Western United States university (Males = 80, Females = 373, Transgender = 1, Nonbinary = 1; *M*age = 20.07, *SD*age = 4.78). Data collection took place in the following semesters: Fall 2020, Spring 2021, Fall 2021, and Spring 2022. Participants were required to be 18 years of age or older. All participants signed up for and completed measures and questionnaires for the study online using the department’s SONA study advertisement system and they received credits they could earn toward courses as part of course requirements or extra credit. Data are cross-sectional – different samples/participants participated in different semesters. Students have to enter a unique SONA ID that never changes when participating in research, meaning that it is impossible for the same participant to complete the study twice.

### Measures

#### HEXACO personality inventory revised

The HEXACO personality inventory revised (HEXACO-PI-R) (Ashton and Lee, 2009) is a 60-item personality measure scored on a 5-point Likert scale (1 = *Strongly Agree* to 5 = *Strongly Disagree*) designed to assess six distinct personality traits: Honesty-humility, emotionality, extraversion, agreeableness, conscientiousness, and openness. The HEXACO-PI-R is an extension of previous research done on the Big 5 constructs of personality (openness, conscientiousness, extraversion, agreeableness, neuroticism). The HEXACO model and Big 5 model contain the same traits excepting that aspects of what is previously labeled as “neuroticism” have been labeled as “emotionality” and the concept of trait honesty-humility has been added to the HEXACO. Some changes worth being noted are that Emotionality has much of the same content that neuroticism (in the Big 5) has, but it lacks the anger-related components of neuroticism and includes the sentimentality component of the Big 5’s agreeableness factor.

The extraversion scale measures social self-esteem, social boldness, sociability, and liveliness. An example item is “I feel reasonably satisfied with myself overall.” The Cronbach’s alpha = 0.78.

The agreeableness scale measures forgiveness, gentleness, flexibility, and patience. An example item is, “I rarely hold a grudge, even against people who have badly wronged me.” The Cronbach’s alpha =0.73.

The conscientiousness scale measures organization, diligence, perfectionism, and prudence. An example item is, “I plan ahead and organize things, to avoid scrambling at the last minute.” The Cronbach’s alpha =0.76.

The emotionality scale measures fearfulness, anxiety, dependence, and sentimentality. An example item is, “I would feel afraid if I had to travel in bad weather conditions.” The Cronbach’s alpha = 0.77.

The honesty-humility scale measures sincerity, fairness, greed-avoidance, and modesty. An example item is, “I would not use flattery to get a raise or promotion at work, even if I thought it would succeed.” Cronbach’s alpha =0.72.

The openness scale measures aesthetic appreciation, inquisitiveness, creativity, and unconventionality. An example item is, “I’m interested in learning about the history and politics of other countries.” =0.73.

#### Sadism

The Assessment if Sadistic Personality scale (ASP; Plouffe et al., 2017) is a 9-item measure scored on a 5-point Likert scale (1 = *Strongly disagree* to 5 = *Strongly agree*). The ASP measures subclinical sadism and focuses on propensity for subjugation, pleasure-seeking, and lack of empathy. An example item is, “I have made fun of people so that they know I am in control.” Cronbach’s alpha = 0.82.

#### Narcissism

The Grandiose Narcissism Scale (GNS; Foster et al., 2015) is a 33-item measure scored on a 6-point Likert scale (1 = *Strongly disagree* to 6 = *Strongly agree*). The GNS is designed to measure grandiose narcissism and includes six subscales measuring authority, self-sufficiency, superiority, vanity, exhibitionism, entitlement, and exploitativeness. An example item is, “I deserve more out of life than other people.” Cronbach’s alpha = 0.90.

#### Compassionate love for humanity

The Compassionate Love for Humanity scale (CLH; Sprecher and Fehr, 2005) is a 21-item measure scored on a 7-point Likert scale (1 = *not at all true of me* to 7 = *very true of me*). The CLH is designed to assess compassionate love for humanity, including behavior and feelings that revolve around concern, care, and support for strangers when they need help. An example item is, “When I see people I do not know feeling sad, I feel a need to reach out to them.” Cronbach’s = 0.95.

#### Questions about mask wearing, social distancing, and hoarding

Participants were asked about frequency of mask wearing and social distancing as well as whether they engaged in hoarding.

Concerning mask wearing, participants were asked, “How often do you wear a mask when you are around other people in public?” Answer options were “Never,” “Sometimes,” “About half the time,” “Most of the time,” and “Always.” However, because group sizes were drastically different, data were recoded to divide participants into two (instead of five) groups – “Always wore masks” and “Did not always wear masks.” Originally the breakdown of groups was: Never wore mask = 3, Sometimes wore masks = 21, Wore masks about half the time = 18, Wore masks most of the time = 94, Always wore masks = 319. When redistributing participants into two groups, those who always wore masks (*N* = 319) were one group and those in all other groups (*N* = 136) were categorized as not always wearing masks.

Participants were given the same answer options when asked “How often do you socially distance when in public (try to remain at least 6 feet away from others).” There was, again, drastically different group sizes for this question. Because of this, participants were divided into two groups – “Always socially distanced” and “Did not always socially distance.” Originally the breakdown of groups was: Never socially distanced (*N* = 10), Sometimes socially distanced (*N* = 35), Socially distanced about half the time (*N* = 43), Socially distanced most of the time (*N* = 197), Always socially distanced (*N* = 170). When redistributing participants, those who always socially distanced (*N* = 170) remained as one group and all other groups were combined into a group labeled as not always socially distancing (*N* = 285).

Participants were also asked, “During the pandemic, some items have become scarce (not many, if any, on shelves) at one point or another. Did you find yourself buying scarce items if you did not immediately need them within the next 2 weeks because of COVID19?” With answer options of “Yes” (*N* = 262) and “No” (*N* = 193). If they chose yes, an additional question was displayed asking them to choose from a list of products to indicate what items they hoarded (cleaning products, hand sanitizer, personal protection equipment, food items, toilet paper, other [with the option to write other products]).

## Results

### Mask wearing

Participants were divided into two groups – those who reported always wearing masks (*N* = 319; *F* = 272, *M* = 45, T = 1, NB = 1) and those who reported that they did not always wear masks (*N* = 136; *F* = 35, *M* = 35, T = 0, NB = 0). Independent samples *t*-tests were conducted to examine differences between groups in the DVs.

The possibility of obtaining false positives increases with every additional *t*-test performed. Because of this, a Bonferroni correction was applied (Field, 2013). The Bonferroni correction divides the alpha level (0.05) by the number of analyses performed (in this case, 9). The corrected *p-*value at which analyses would be considered significant is 0.006. Following these guidelines, those who reported always wearing masks were significantly higher in compassionate love for humanity and significantly lower in sadism compared to those who did not always wear masks. There were no other significant differences in any other comparisons. See Table 1 for descriptives and inferential statistics information (e.g., *t* values, *p-*values, effect sizes).

**Table 1:** Personality differences between those who always and do not always wear masks.

Trait variable	Always (*M*, *SD*) *N* = 319	Not Always (*M*, *SD*) *N* = 136	*t* value (df)	Two-sided *p*-value	Cohen’s *d*
Openness	33.07 (5.98)	31.62 (5.20)	2.60 (290.88)	0.010	0.25
Conscientiousness	35.73 (5.75)	34.49 (5.39)	2.20 (270.51)	0.028	0.22
Agreeableness	32.60 (5.83)	31.14 (4.97)	2.72 (296.10)	0.007	0.26
Extraversion	29.45 (6.62)	30.05 (5.56)	−0.99 (300.89)	0.323	−0.09
Emotionality	35.37 (6.01)	34.67 (6.41)	1.56 (241.01)	0.120	0.16
Honesty-humility	35.56 (5.90)	34.63 (5.40)	1.64 (277.18)	0.102	0.16
Assessment of Sadistic Personality	14.01 (5.38)	16.08 (5.96)	−3.49 (233.23)	0.001	−0.37
Narcissism total	98.73 (21.72)	102.21 (19.53)	−1.68 (281.96)	0.095	−0.17
Compassionate love for humanity	98.05 (22.61)	91.32 (22.51)	2.92 (255.98)	0.004	0.30

Statistics are reported from Equal Variances Not Assumed information due to the difference in size of groups.

### Social distancing

Participants were divided into two groups – those who always socially distanced (*N* = 170; *F* = 142, *M* = 27, T = 1, NB = 0) and those who did not always socially distance (*N* = 285; *F* = 231, *M* = 53, T = 0, NB = 1). Independent samples t-tests were conducted to examine differences between groups in the DVs.

After conducting a Bonferroni correction with a new required *p-*value of 0.006, those who reported always socially distancing were significantly higher in trait openness, conscientiousness, and compassion for others compared to those who did not always socially distance. See Table 2 for descriptives and inferential statistic information (e.g., *t* values, *p*-values, effect sizes).

**Table 2:** Personality differences between those who always and did not always socially distance.

Trait variable	Always socially distanced (*M*, *SD*) *N* = 170	Did not always socially distance (*M*, *SD*) *N* = 285	*t* value (df)	Two-sided *p*-value	(Cohen’s *d*)
Openness	33.69 (5.53)	32.00 (5.86)	3.09 (371.88)	0.001	0.30
Conscientiousness	36.53 (5.87)	34.67 (5.43)	3.37 (333.82)	0.001	0.33
Agreeableness	32.51 (5.83)	31.96 (5.49)	1.00 (338.49)	0.317	0.10
Extraversion	29.24 (6.90)	29.87 (5.95)	−0.98 (315.16)	0.326	−0.10
Emotionality	35.08 (6.46)	35.55 (5.95)	−0.77 (322.80)	0.445	−0.08
Honesty-humility	36.18 (6.10)	34.75 (5.50)	2.50 (326.54)	0.013	0.25
Assessment of sadistic personality	14.22 (5.39)	14.88 (5.77)	−1.23 (374.67)	0.220	−0.12
Narcissism total	98.39 (22.14)	100.60 (20.49)	−1.06 (334.21)	0.290	−0.11
Compassionate love for humanity	100.06 (24.04)	93.64 (21.66)	2.86 (326.66)	0.005	0.28

Statistics are reported from Equal Variances Not Assumed information due to the difference in size of groups.

### Hoarding

Participants were divided into two groups – those who hoarded (*N* = 262; *F* = 220, *M* = 42, *T* = 0, NB = 0) and those who did not hoard (*N* = 193; *F* = 153, *M* = 38, T = 1, NB = 1). Independent samples *t*-tests were conducted to examine differences between groups in the DVs.

Of those who reported hoarding, toilet paper was listed as the most hoarded item (*N* = 148). Other common items were food items (*N* = 134), cleaning products (*N* = 125), hand sanitizer (*N* = 102), and personal protection equipment (*N* = 91). After conducting a Bonferroni correction with a new required *p* value of 0.006, it was found that those who did not hoard were significantly higher in agreeableness than those who did hoard. There were no other significant differences in any traits. See Table 3 for descriptives and inferential statistic information (e.g., *t* values, *p*-values, effect sizes).

**Table 3:** Personality differences between those who did and did not hoard.

Trait variable	Hoarded (*M*, *SD*) *N* = 262	Did not hoard (*M*, *SD*) *N* = 193	*t* value (df)	Two-sided *p*-value	Cohen’s *d*
Openness	32.92 (5.85)	32.24 (5.69)	1.25 (420.11)	0.210	0.12
Conscientiousness	35.69 (5.52)	34.92 (5.84)	1.43 (400.48)	0.154	0.14
Agreeableness	31.53 (5.51)	33.02 (5.66)	−2.80 (407.66)	0.005	−0.27
Extraversion	29.48 (6.56)	29.83 (5.99)	−0.59 (432.55)	0.555	−0.06
Emotionality	35.88 (5.73)	34.68 (6.62)	2.02 (377.59)	0.044	0.20
Honesty-humility	35.16 (5.71)	35.46 (5.85)	−0.54 (407.89)	0.591	−0.05
Assessment of sadistic personality	14.53 (5.58)	14.76 (5.72)	−0.42 (407.97)	0.672	−0.04
Narcissism total	99.85 (20.22)	99.67 (22.34)	0.09 (387.09)	0.928	0.10
Compassionate love for humanity	95.60 (23.00)	96.63 (22.49)	−0.48 (418.88)	0.634	−0.05

Statistics are reported from Equal Variances Not Assumed information due to the difference in size of groups.

## Discussion

Past research on the Big 5 and HEXACO (e.g., Carvalho et al., 2020; Xie et al., 2020; Airaksinen et al., 2021; Götz et al., 2021; Han, 2021; Costantini et al., 2022) has shown a pattern wherein higher levels of openness, conscientiousness, neuroticism/emotional stability, honesty-humility, and agreeableness predict a greater likelihood to wear masks and socially distance. Past research has also shown that higher levels of extraversion predict a decreased likelihood for things like socially distancing. The current research also found differences in traits such as agreeableness, openness, and conscientiousness between those who did and did not consistently wear masks and socially distance. However, there were no significant differences between groups in extraversion, honesty-humility, or emotionality (neuroticism/emotional stability), thus the current research failed to replicate past studies or support hypotheses of the current study concerning these variables.

The current study also replicated past research (Fazio et al., 2021; Karnaze et al., 2022) on compassion for others; those who always wore masks in the current study were significantly higher in compassion for others.

Past research (Triberti et al., 2021; Chávez-Ventura et al., 2022) has found that dark triad traits such as narcissism and sadism predict the propensity to mask and socially-distance. The current study somewhat replicated past research by showing that those who did not always mask were significantly higher in sadism compared to those who did always mask.

Personality factors and hoarding have not been widely studied. Findings of the current study did not fully support past research (Yoshino et al., 2021). Although the current study found that those who hoarded were significantly lower in agreeableness (similar to Yoshino et al., 2021) compared to those who did not hoard, hypotheses that those who hoarded would be higher in neuroticism/emotional stability, narcissism, sadism and lower in honesty-humility, and compassion for others supported.

Although the current study focuses on existing, unaltered trait levels of personality characteristics, past research (Rieger, 2020) from earlier in the pandemic has shown that inducing a prosocial mindset can increase the self-reported likelihood of engaging in behaviors that protect others. Rieger (2020) primed people with reasons for getting vaccinated, including two selfish motivations (emphasizing a personal risk or the inconvenience of infection to the participant) or one altruistic reason (protecting others who are vulnerable or cannot be vaccinated). Although all prompts were successful at increasing the self-reported likelihood of being vaccinated, the prompt that made the protection of others more salient was significantly more effective.

Unlike some past research (e.g., Carvalho et al., 2020; Götz et al., 2021; Han, 2021) there were no significant differences in extraversion in any of the categories. One possible explanation is that much of the previous research on personality and pandemic-related behavior was conducted rather early in the pandemic, while the current study took place in university students across several semesters starting in Fall 2020 and ending in Spring 2022. It might be conceivable that, as the pandemic went on, even those with higher levels of extraversion and therefore a higher drive to socialize would eventually realize the severity of the situation and rein in their social impulses. Another possible explanation may be the types of participants used in previous studies. For example, Götz et al. (2021) and Han (2021) used large-scale international datasets and Carvalho et al. (2020) also used a general population sample. A limitation of the current study is that data were collected from university students, specifically from a participant pool of students taking psychology courses. It might be possible that influence from the university stressing the severity of the pandemic incited a sense of urgency and importance on sheltering-in-place that overrode the extraverted desire to seek out and spend time with others.

In addition to the sample comprising of students, other limitations are that the current study did not manipulate state levels of any of the traits examined to determine whether manipulations might influence self-reported likelihood to hoard, socially distance, or wear masks. The study would have been strengthened by having inductions designed to temporarily increase things like conscientiousness, agreeableness, honesty-humility, compassion for others, and temporarily decrease narcissism, sadism etc. and then giving participants scenarios wherein one should mask, socially distance, and where one might want to hoard and ask how participants might behave in such situations. Because there were no experimental manipulations, it is difficult to determine whether the traits measured actually influence pandemic-related behaviors.

Another limitation is that 82% of the participants are female. Data were collected mainly from students in psychology courses and research (Willyard, 2011) that women make up roughly 76% of psychology majors. There may have been gender bias in results as past research has shown that women tend to be lower in dark triad type traits (e.g., Jonason and Davis, 2018). Schmitt et al. (2008) also found that women tend to be higher in conscientiousness and agreeableness than men. Women also tend to have higher levels of compassion for others (Beresford, 2016).

The location of the study may have played an important role, also. Specifically, the way the local government handled the pandemic and the type of information it gave its citizens may have impacted behaviors. Despite the objective severity of the virus, research (Bursztyn et al., 2020) has shown that society-wide issues such as misinformation from local governments surrounding the COVID-19 can impact safety and preventative measures on the part of citizens, potentially resulting in excess and preventable deaths compared to areas of the country with less widespread misinformation. The location of the current study was in California, a more “liberally” minded state in the U.S. that exercised extreme caution concerning health and safety during the pandemic compared to some other states. Locations with more collectivistic mindsets (vs. individualistic) had more prosocial behaviors and better health outcomes (Shekriladze et al., 2021). Findings that collectivistic cultures may be more prosocial may explain why prosocial traits played an important role in the current study – especially in light of the fact that women comprised the majority of participants and also tend to be higher in prosocial traits.

Another limitation is that people could be over-reporting positive prosocial behaviors and under-reporting negative behaviors. For example, Gosling et al. (1998) found that, when asked to self-report on one’s own behaviors related to traits, only 19% of participants accurately reported their behavior with 57% of participants over-reporting desirable acts and 24% under-reporting undesirable acts.

## Conclusion

In general, findings from the current study suggest that prosocial traits are related to greater self-reported behaviors that involve protecting the self and others, whereas darker traits such as sadism and aspects of narcissism are related to behaviors that can potentially negatively impact the self and others. Implications of the current study are that naturally occurring differences in consideration and compassion for others may play a role in prosocial behaviors that affect those around us. Because of this, finding ways to increase such caring behaviors in others may create a healthier society. Considering that COVID-19 still poses a global problem and that many economies are re-opening and cutting back on mandates to shelter in place as well as shortening quarantine time, emphasizing the importance of protecting others may be a needed step in decreasing preventable deaths. For example, future research could expand on that of Rieger (2020) to increase a sense of altruism via community-based participatory avenues with the goal of increasing vaccination and booster rates to protect not only the self, but also others. Currently, in the United States, it is estimated that 79% of the population has had at least one vaccine dose, only 68% of people are considered fully vaccinated, and only 33% of people have had a booster dose (USA Facts, 2023). While it may no longer be feasible to quarantine and shelter in place due to the capitalist drive to “re-open economies,” it might be beneficial to encourage focus on state boosts of characteristics such as agreeableness, compassion for others, etc. in order to increase vaccination and booster rates. For example, physicians may use this information when providing education to clients during check-ups and in-clinic visits. Physicians could attempt to temporarily increase altruistic-type behaviors during visits by elaborating on how vaccinations protect friends, loved ones, and the community to encourage first-time vaccinations as well as boosters. Discussing the impact of low vaccination rates on vulnerable populations may also temporarily increase altruistic-related behaviors that encourage patients to receive vaccines.

## Data availability statement

The raw data supporting the conclusions of this article will be made available by the authors, without undue reservation.

## Ethics statement

The studies involving humans were approved by California State University, Bakersfield Human Subjects IRB. The studies were conducted in accordance with the local legislation and institutional requirements. The participants provided their written informed consent to participate in this study.

## Author contributions

JW: Conceptualization, Data curation, Formal analysis, Investigation, Methodology, Project administration, Resources, Software, Supervision, Validation, Visualization, Writing – original draft, Writing – review & editing.
